# Generating and measuring effective vaccine-elicited HIV-specific CD8^+^ T cell responses

**DOI:** 10.1097/COH.0000000000000824

**Published:** 2023-09-20

**Authors:** Gina M. Borgo, Rachel L. Rutishauser

**Affiliations:** Department of Medicine, University of California, San Francisco, California, USA

**Keywords:** CD8^+^ T cell, HIV vaccine, nucleic acid vaccine platforms, T cell quality, viral vectors

## Abstract

**Purpose of review:**

There is growing consensus that eliciting CD8^+^ T cells in addition to antibodies may be required for an effective HIV vaccine for both prevention and cure. Here, we review key qualities of vaccine-elicited CD8^+^ T cells as well as major CD8^+^ T cell-based delivery platforms used in recent HIV vaccine clinical trials.

**Recent findings:**

Much progress has been made in improving HIV immunogen design and delivery platforms to optimize CD8^+^ T cell responses. With regards to viral vectors, recent trials have tested newer chimp and human adenovirus vectors as well as a CMV vector. DNA vaccine immunogenicity has been increased by delivering the vaccines by electroporation and together with adjuvants as well as administering them as part of a heterologous regimen. In preclinical models, self-amplifying RNA vaccines can generate durable tissue-based CD8^+^ T cells. While it may be beneficial for HIV vaccines to recapitulate the functional and phenotypic features of HIV-specific CD8^+^ T cells isolated from elite controllers, most of these features are not routinely measured in HIV vaccine clinical trials.

**Summary:**

Identifying a vaccine capable of generating durable T cell responses that target mutationally vulnerable epitopes and that can rapidly intercept infecting or rebounding virus remains a challenge for HIV. Comprehensive assessment of HIV vaccine-elicited CD8^+^ T cells, as well as comparisons between different vaccine platforms, will be critical to advance our understanding of how to design better CD8^+^ T cell-based vaccines for HIV.

## INTRODUCTION

The majority of vaccines being developed for HIV prevention aim to elicit antibody responses against the virus, ideally broadly neutralizing antibodies (bNAbs) that can recognize diverse Env sequences [1]. Although there is strong evidence that bNAbs can protect from neutralization-sensitive viral infection in preclinical and clinical studies [2], and while there has been considerable progress towards this goal in recent years, no HIV vaccine strategy to date has successfully generated high titers of HIV bNAbs. T cells, specifically CD8^+^ T cell responses, can contribute to control of HIV infection [3–5] and therefore may be useful to target in the context of both preventive and therapeutic HIV vaccines. Unlike neutralizing antibodies, virus-specific CD8^+^ T cells can directly kill infected cells [6]. Additionally, they may offer an added layer of immunity in cases where antibodies are not fully protective [7^▪▪^,^8^], they may provide more robust protection against antigen escape (i.e., broader antigen coverage) [9–11], and they may amplify activation and recruitment of other cell types to sites of infection [12].

In this review, we will describe our understanding of ideal features required for HIV vaccine-elicited CD8^+^ T cells and what is known about the CD8^+^ T cell immunogenicity of current vaccine platforms that seek to elicit robust virus-specific CD8^+^ T cell responses. We will not focus on immunogen design, as that has been covered in depth in recent reviews [13,14^▪^,15^▪^]. We will discuss methods to comprehensively measure the quality of vaccine-elicited CD8^+^ T cell responses and, finally, we will consider lessons from HIV therapeutic vaccine studies that may inform prevention strategies. 

**Box 1:**
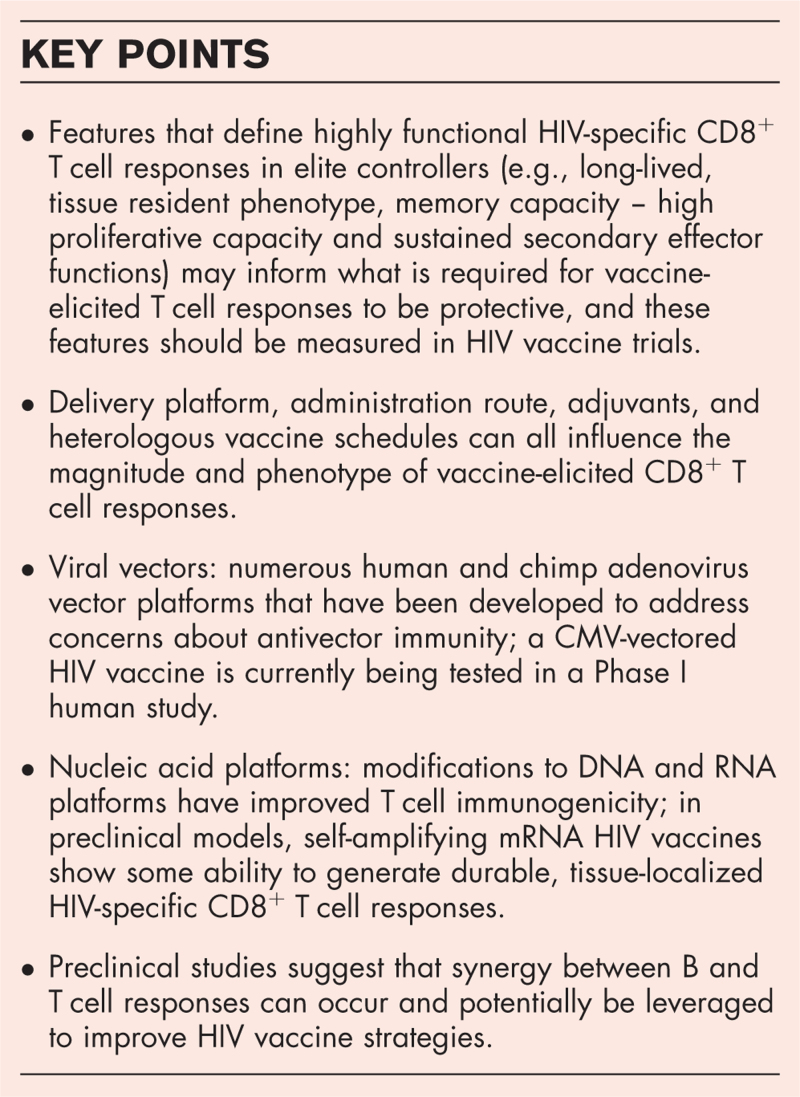
no caption available

## THE ULTIMATE GOAL: WHAT FEATURES DEFINE AN EFFECTIVE HIV-SPECIFIC CD8^+^ T CELL RESPONSE?

Although most individuals with HIV generate HIV-specific CD8^+^ T cell responses early in infection [3,5,6,16,17], the majority of people with HIV cannot control viremia without antiretroviral therapy (ART). Rare individuals known as elite controllers [<1% of people with HIV (PWH)] do control viremia to undetectable levels in the absence of ART, and several lines of evidence suggest a role for CD8^+^ T cells in establishing and maintaining this control [3,4,18^▪▪^,^19^–^21^]. Direct control of infection by CD8^+^ T cells has been demonstrated by experiments in simian immunodeficiency virus (SIV)- or simian-human immunodeficiency virus (SHIV)-infected nonhuman primates (NHPs) in which CD8α or CD8β depletion led to an increase in viral load [22–27]. Finally, a rhesus cytomegalovirus (RhCMV)-vectored vaccine that elicits CD8^+^ T cells but no antibody responses has been shown to prevent the establishment of chronic SIV infection in nearly 60% of vaccinated animals [28,29^▪▪^,^30^,31^▪^]. Therefore, CD8^+^ T cells can, at least in some settings, contribute to control of retroviral infection.

**FIGURE 1:**
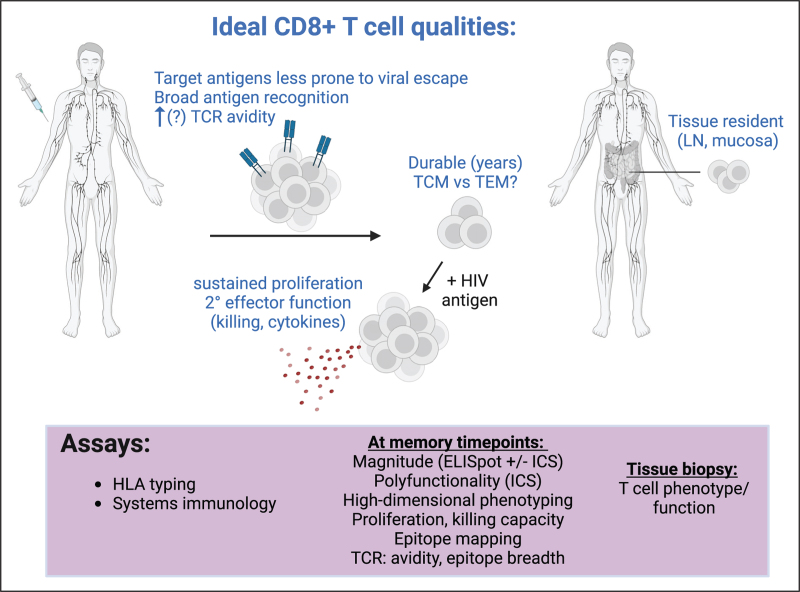
Ideal qualities of vaccine-elicited CD8^+^ T cell responses (in blue) and assays to comprehensively measure these qualities (purple box). LN, lymph node; TCM, central memory; TCR, T cell receptor; TEM, effector memory.

Based on studies in natural HIV/SIV infection and from preclinical testing of HIV vaccine candidates, we believe that successful control of HIV by vaccine-elicited CD8^+^ T cells will likely require that the CD8^+^ T cells have the following features (see Fig. 1):

(1) target viral epitopes that are less likely/unable to be mutated and likely target a broad range of these epitopes across HLA types [13,14^▪^,15^▪^,^32^,33^▪^],
(2) express T cell receptors (TCRs) with broad epitope reactivity [34,35] and optimal avidity (in some settings, low avidity may enable cross reactivity [36], while in others high avidity may be important for T cell cytotoxic function [34,37]),
(3) are durably maintained at a high magnitude at relevant sites of infection (e.g., gut, rectal, and vaginal mucosa, as well as lymphoid tissue) [18^▪▪^,^38^,^39^], and
(4) occupy a memory-like differentiation state that allows them to robustly proliferate [40^▪^] and acquire effector functions (e.g., cytotoxicity, cytokine production) upon encountering antigen [20,21,38,41,42].

While many of these features are well defined in the setting of natural HIV infection or preclinical animal models, less is known about how they actually relate to the protective capacity of HIV-specific CD8^+^ T cells elicited by HIV vaccines administered in people. One clinical trial, HVTN 505 (DNA/Ad5), did report a correlation between Env-specific CD8^+^ T cell magnitude and polyfunctionality and decreased infection risk (hazard ratio = 0.51 and 0.47, respectively) [43,44]. With regards to epitope targeting, earlier HIV vaccine inserts typically encoded full-length viral proteins, but it is now clear that more narrowly targeting evolutionarily conserved and/or structurally constrained epitopes/regions more efficiently elicits CD8^+^ T cell responses that are predicted to be less likely to be evaded by viral mutation [13,14^▪^,15^▪^,^32^,33^▪^,^45^,^46^^▪▪^,^47^]. Some specific HLA class I alleles have been associated with elite controller status or altered rates of disease progression [19,48–50]. Mamu type-specific effects on vaccine protection have been observed in NHPs [51,52] and HLA-adaptation of T cell epitopes may impact vaccine-elicited T cell responses in people [53^▪▪^]. In terms of differentiation state, it is unclear which specific differentiation state(s) will be most beneficial/critical to elicit in the context of a preventive vaccine for HIV. Virus-specific memory CD8^+^ T cells in elite controllers express high levels of the T cell memory-associated transcription factor, TCF-1, and are highly proliferative upon antigen encounter [40^▪^,^54^,^55^]. On the other hand, SIV-specific MHC-E restricted CD8^+^ T cells with an effector memory phenotype are the predominant subset elicited by protective RhCMV-vectored vaccines [29^▪▪^,^30^,31^▪^,56^▪^]. As we discuss in the latter section of this review, comprehensive evaluation of all of the HIV-specific CD8^+^ T cell properties depicted in Fig. 1 will be required to meaningfully compare how different CD8^+^ T cell-based HIV vaccine platforms elicit them and how they in turn relate to immune protection.

## T CELL-BASED VACCINE DELIVERY PLATFORMS

The choice of vaccine delivery platform (e.g., protein, nucleic acid, viral vector) and route of administration determines how immunogens are presented, in what tissues, and for how long, and thus significantly impacts the immunogenicity and durability of vaccine-elicited immune responses [57,58,59^▪^]. Vaccine platforms that use protein/subunit (AIDSVAX), viral vector (Ad5, ALVAC-HIV, Ad26, MVA), and plasmid DNA (DNA-HIV-PT123, VRC-HIVDNA009-00-VP) have been used in HIV vaccine efficacy trials. Because viral vector and nucleic acid-based delivery platforms can elicit robust T cell responses (unlike protein-based vaccines) [60], we will review what is known about the antigen-specific CD8^+^ T cell responses elicited by these different vaccine approaches based on recent human HIV vaccine clinical trials in HIV (see Table 1) and other contexts.

**Table 1:** Summary of HIV preventive vaccine strategies aimed at eliciting CD8^+^ T cells

Year results published	Trial name	NCT #	Phase	Delievery	Vaccine	Immunogen design	% with CD8 response	Notes	Publications; trials (cure)
2008	HVTN 502, STEP, Merck 023	NCT00095576	2	Ad5, IM	MRKAd5 HIV-1	Full sequence consensus; Gag/Pol/Nef (clade B)	73%	4 w post-boost; higher rates if low Ad5 titers	[65,67]
2011	HVTN 204	NCT00125970	2	Prime: DNA, IM/EP Boost: Ad5, IM	VRC DNA/rAd5	Full sequence consensus; Gag/Pol/Nef (clade B) and Env (clades A, B, C)	47%	6 w post-boost	[134] Cure: [135]
2013	HVTN 505	NCT00865566	2	Prime: DNA, IM/biojector Boost: Ad5, IM	VRC DNA/rAd5	Full sequence consensus; Gag/Pol/Nef (clade B) and Env (clades A, B, C)	64%	4w post-boost	[83] Cure: [135]
2013	HVTN 080, PENNVAX	NCT00991354	1	DNA, IM/EP	PENNVAX®-GP HIV-1 DNA vaccine +/- IL-12 DNA	Full sequence consensus; Gag, Pol, and Env (clade B)	52%	2w post-3rd dose (+IL-12+EP group)	[136] Cure: [133], NCT03606213
2014	HIV-CORE-002	NCT01151319	1	Prime: ChAdV63/Boost: MVA; IM. Prime: DNA/Boost: ChAdV62, MVA; IM. Prime: DNA/Boost: MVA, ChAdV62; IM	ChAdV63.HIVconsv, pSG2.HIVconsv, MVA.HIVconsv	Conserved region/consensus sequence; Gag (clades A, C, D), Pol (clades A, B, C, D), Vif (clade D), Env (clades C, D)	response rates not reported	n/a	[13,47,114] Cure (immunogen): [137–140]
2017	HVTN 087	NCT01578889	1	Prime: DNA, IM/EP Boost: VSV, IM	ProfectusVax: HIV-MAG +/- IL-12 DNA + VSV-Gag	Full sequence consensus; Gag/Pol/Nef/Tat/Vif/Env (clade B)	49%	6 m post-boost; all participants +IL-12	[108,112] Cure (HIV-MAG): [141]
2019	HVTN 098, PENNVAX	NCT02431767	1	DNA, ID or IM [EP]	PENNVAX®-GP HIV-1 DNA vaccine +/- IL-12 DNA	Full sequence consensus; Gag (clades A, B, C, D), Pol, and Env (clades A, C)	65% (ID), 54% (IM)	6 m post-boost; +IL-12 group (lower without)	[109^▪▪^,^142^] Cure: [133], NCT03606213
2020	HVTN 117, TRAVERSE	NCT02788045	1/2	Prime: Ad26, IM Boost: protein, IM	Ad26.Mos.HIV+Clade C gp140; Ad26.Mos4.HIV+Clade C gp140	Mosaic; Gag/Pol (based on group M), and Env (clades B, C, CRF01_AE; for Mos4)	33%	6 m post-boost; Gag-specific (tetravalent)	[91] Cure (MVA boost): [143]
2021	HVTN 106	NCT02296541	1	Prime: DNA, IM Boost: MVA, IM	DNA Nat-B env or DNA CON-S env or DNA mosaic env plus MVA-CMDR boost	Natural isolate, consensus or mosaic; All express: gp160 Env Nat-B (Clade B), Con-S and mosaic: Env for group M; MVA-CMDR: Env/Gag/Pol (clades A and E)	29% (Nat-B), 36% (Con-S), 22% (mosaic)	6m post-boost	[46^▪▪^,^144^]
2023	HVTN 118, ASCENT	NCT02935686	1/2	Prime: Ad26, IM Boost: protein, IM	Ad26.Mos4.HIV+Clade C gp140; Ad26.Mos4.HIV+Clade C gp140 +Mosaic gp140	Mosaic; Gag/Pol (group M), and Env (clades B, C, CRF01_AE); Mosaic gp140 (group M)	18%	6m post-boost; Gag-specific (mosaic group)	[145^▪^] Cure (Ad26.Mos4): NCT04983030
2023	HVTN 112	NCT02654080	1	Prime: DNA, IM/EP Boost: VSV, IM	HIV-1 nef/tat/vif, env pDNA vaccine + rVSV HIV envC	Natural isolate; Nef/Tat/Vif (clade B) and Env (clades B and C)	18%, 0%	2w post-boost (1st, 2nd); Env-specific	[146]
2023 (halted)	HVTN 706, Mosaico	NCT03964415	3	Prime: Ad26, IM Boost: protein, IM	Ad26.Mos4.HIV + Clade C gp140 + Mosaic gp140	Mosaic; Gag/Pol (group M), and Env (clades B, C, CRF01_AE); Mosaic gp140 (group M)			Cure (Ad26.Mos4): NCT04983030
									
TBD	HIV-CORE-006	NCT04553016	1	Prime: ChAdOx1, IM; Boost: MVA, IM	ChAdOx1.tHIVconsv1, MVA.tHIVconsv3, MVA.tHIVconsv4	Conserved/mosaic; Gag/Pol (group M)			Immunogen: [47,114] Cure (immunogen): [147], NCT03844386
TBD	HIV-CORE-0051	NCT04563377	1/2a	Prime: ChAdOx1, IM; Boost: MVA, IM	ChAdOx1.HTI, MVA.HTI	T cell responses associated with viral control in PWH; Gag, Pol, Nef, Vif (clades B and C)			Preclinical: [14^▪^,^32^] Cure: [129,148], NCT04364035
TBD	VIR-1111	NCT04725877	1	Human CMV, SC	VIR-1111	UD			
TBD	HVTN 119	NCT03181789	1	DNA, IM/EP	p24CE1/2 pDNA + p55^gag +IL-12 DNA	Conserved elements; Gag p24, p55 (group M)			Preclinical: [149] Cure: NCT03560258

EP, electroporation; ID, intradermal; IM, intramuscular; SC, subcutaneous; UD, undisclosed.

### Viral vector vaccines

Viral vectors have been a consistent part of the HIV vaccine pipeline including in the RV144 trial [61–64], designed to elicit antibody responses, and STEP/Phambili trials, designed to elicit CD8^+^ T cell responses [65–67]. Viral vectors can generate durable T cell responses without the need for an adjuvant [68,69] and can be administered intranasally and orally to specifically target mucosal responses [68,70–74]. Recent and currently active HIV preventive vaccine trials utilize poxvirus viral vectors [modified vaccinia virus Ankara (MVA)], human (Ad4, Ad26) and chimp (AdC6, AdC7, ChAdOx1) adenoviruses, and human cytomegalovirus. Additional viral vectors have been used in other vaccine settings, with the most detailed description of the magnitude, durability, and memory-like qualities of the response being described for the live-attenuated Yellow Fever Vaccine [75–77].

In general, human adenovirus vectors can elicit robust CD8^+^ T cell responses [68,69]. The human adenovirus vector, Ad5, was the first viral vector to be tested in efficacy trials for HIV (STEP trial/MRKAd-5 HIV), specifically with the goal of eliciting CD8^+^ T cell responses that target Gag/Pol/Nef [65]. In this trial, nearly 75% of vaccinated participants tested formed detectable HIV-specific T cell responses in response to vaccination as measured by interferon gamma (IFNγ) ELISpot 4 weeks after the last dose [67]. Although the vaccine did not generally elicit a broad CD8^+^ T cell response [78] and was not protective (vaccinated men who were Ad5 seropositive and uncircumcised had transient increased rates of infection [65,67]), there was an association between vaccine-generated responses to three or more Gag epitopes and reduced viral loads [43]. Much follow-up work has been done to understand the increased risk and overall outcomes of the STEP trial [53^▪▪^,^79^,^80^]. Ad5 continues to be used in heterologous vaccine approaches [81–85]. Other human adenoviruses, Ad26 and Ad35, have also been used due to lower preexisting immunity [86–88]. Preclinical studies in the context of HIV and other settings demonstrate that, compared with Ad5, these vectors generate CD8^+^ T cell responses at lower magnitude [68,69,86–89], but they may generate responses with improved T cell memory properties (e.g., long-lived Ad26-elicited CD8^+^ T cells have a more terminally phenotype compared to Ad5-elicited T cells) [87–90]. Ad26 expressing mosaic Gag/Pol/Env immunogens with bivalent Env (clade C/mosaic gp140) protein boost was recently tested in the Mosaico phase III trial (HVTN 706/NCT03964415). Previous trials that utilized earlier iterations of the vaccines used in Mosaico elicited Gag-specific CD8^+^ T cell responses in 32% (tetravalent [Gag/Pol/Env1/Env2] Ad26 mosaic design) 6 months after the last dose [91]. Mosaico was stopped in early 2023 due to lack of efficacy at preventing HIV infection.

Chimp adenovirus vectors have also been developed to avoid preexisting vector immunity to human adenovirus vectors [92,93] and two chimp adenovirus vectors, ChAdOx1 and AdC6/AdC7, are currently being utilized in phase I clinical trials for HIV (via intramuscular injection; NCT04553016, NCT05182125). In a side-by-side comparisons of chimp to human adenovirus vectors in mice, human Ad5 and chimp Ad3 showed equivalent Gag-specific CD8^+^ T cell response magnitude (as measured by MHC class I tetramer staining) and protective capacity upon challenge with *Listeria monocytogenes* expressing SIV Gag [87]. HIV-CORE-002 examined the use of heterologous combinations of ChAdOx63, DNA, and MVA to deliver the Gag/Pol/Vif/Env-containing HIVconsv immunogen in volunteers without HIV and found that 100% of participants generated HIVconsv-specific T cell responses following boost as detected by IFNγ ELISpot for all heterologous vaccine schedules tested [47]. Although relatively new to the HIV vaccine pipeline (HIV-CORE-006, HIV-CORE-051), the ChAdOx1 vector developed by Oxford University/AstraZeneca has recently been widely tested and deployed for SARS-CoV-2 (AZD1222) [94]. After a single dose of the ChAdOx1 vaccine, SARS-CoV-2-specific CD8^+^ T cells expressing any combination of the cytokines IFNγ, IL-2, and/or TNFα, as identified by intracellular cytokine staining (ICS), were present at approximately 0.1% of total CD8^+^ T cells 14 days following the vaccine [95]. Compared with lipid nanoparticle (LNP)-formulated mRNA or heterologous (mRNA+ChAdOx1) vaccine approaches, two doses of the ChAdOx1 vaccine elicited a lower overall magnitude of total T cell responses as measured by IFNγ ELISpot [96^▪▪^,^97^,^98^].

The first phase I trial using a human CMV (hCMV) viral vector was recently completed by Vir Biotechnology (NCT04725877), with initial reports indicating that the vaccine is well tolerated [99]. There are several potential advantages of using a CMV vector-based platform to elicit HIV-specific CD8+ T cell responses [29^▪▪^]. First, based on extensive work on rhCMV strain RhCMV68-1, vaccines with RhCMV68-1 expressing SIV immunogens elicited high magnitude, broad effector memory (TEM)-skewed CD8^+^ T cell responses in the absence of an antibody response in 100% of animals, and demonstrated arrest and clearance of SIV in nearly 60% of vaccinated rhesus macaques, with similar efficacy maintained in CMV seropositive animals [28,29^▪▪^,^30^,31^▪^,56^▪^,^100^]. Second, the RhCMV68-1 vaccine generates unconventional MHC-E-restricted HIV-specific CD8^+^ T cells [31^▪^,56^▪^,^101^]. MHC-E is highly conserved and has limited polymorphism compared to classical MHC-I, thus potentially increasing the likelihood that conserved epitopes could be found when adapting the CMV platform for use in humans [29^▪▪^,^102^]. One outstanding question is whether a human CMV vector containing HIV immunogens has the same capacity to generate unconventional MHC-E-restricted responses, and, ultimately whether these responses can prevent the establishment of chronic HIV infection in humans. Furthermore, while MHC-E-restricted responses can be primed *in vitro*[103], it is unknown how they may synergize with conventional MHC class I-restricted CD8^+^ T cell responses and/or other cell types in mediating protection.

### Nucleic acid based vaccines

Nucleic acid-based delivery systems (DNA and RNA) offer distinct advantages over viral vectors: they are less expensive and easier to design/manufacture and they circumvent issues with vector immunity and vector backbone immunogenicity [59^▪^,^60^,^104^]. Whereas hundreds of millions of doses of mRNA vaccines for SARS-CoV-2 have now been administered in humans, DNA vaccines remain in more limited use, despite extensive testing in clinical trials for both cancer and HIV [60,105,106].

Since the time of the first clinical trial to test a DNA vaccine in humans (an HIV therapeutic vaccine) [107], the immunogenicity of DNA-based vaccines has improved with delivery via electroporation and design of regimens that include boosting with a viral vector [104,108,109^▪▪^,^110^–^113^]. Using inserts targeting Gag and Pol consensus sequences, the PENNVAX-GP DNA vaccine (HVTN 098) demonstrated the ability of a DNA vaccine alone [delivered via intramuscular (i.m.) or intradermal injection with plasmid IL-12 adjuvant] to elicit CD4^+^ (96%) and CD8^+^ (44% i.m., 64% intradermal) T cell responses as well as antibody responses (14% i.m., 56% intradermal) 2 weeks after the final dose [109^▪▪^]. When comparing different delivery platforms/vaccination schedules utilizing the HIVconsv vaccine insert, DNA prime plus ChAdV63/MVA boost compared with ChAdV63 prime plus MVA, all vaccinees from both vaccine schedules maintained T cell responses as detected by ELISpot two years postvaccination and the magnitude of these responses was not significantly different between the two vaccine schedules [13,114].

mRNA/LNP-based vaccines saw widespread administration for SARS-CoV-2 and two active phase 1 trials are examining the ability of mRNA vaccines to generate bNAbs to HIV Env (NCT05217641, NCT05001373). In the context of SARS-CoV-2, mRNA/LNP vaccinees elicit memory CD8+ T cell responses in approximately 40–60% of vaccinees 6 months after the second dose [11,115,116], and Spike-specific CD8^+^ T cells are predominantly TEM phenotype, although a stable pool of polyfunctional stem-like memory cells (CD45RA+ CD27+ CD28+ CCR7+ CD95+) with high proliferative capacity can also be detected at long as 9 months after the second dose [11,117^▪^,^118^^▪▪^,^119^]. For individuals who were vaccinated with mRNA/LNP or ChAdOx1 and who subsequently experienced breakthrough infection, the frequency of activated SARS-CoV-2 Spike-specific CD8^+^ T cells at symptom onset inversely correlated with viral clearance [118^▪▪^]. In addition to SARS-CoV-2 vaccines, cancer therapeutic vaccines have specifically sought to optimize CD8^+^ T cell responses using mRNA platforms [120,121]). Recent preclinical studies are utilizing mRNA as a heterologous boost with DNA [122], and self-amplifying RNA (saRNA) [123^▪▪^] and circular RNA [124] also demonstrate the potential of RNA-based platforms in eliciting CD8^+^ T cell responses. Specifically, saRNA delivery of the tHIVconsvX immunogen generated both effector and central memory phenotype CD8^+^ T cells responses that maintained polyfunctionality and proliferative capacity for 22 weeks postvaccination in mice [125], suggesting that this platform may be an effective approach to improving the durability of tissue-localized responses.

## LABORATORY ASSESSMENT OF VACCINE-ELICITED CD8^+^ T CELLS

Aside from what we have discussed above, relatively little is known about how different vaccine approaches (for HIV or in other contexts) influence the quality of the vaccine-elicited T cell responses on people. This gap in our knowledge exists for many reasons, including the fact that very few controlled studies have been designed to test different vectors [47,126,127], adjuvants [128], and/or immunogens [46^▪▪^] side-by-side in well matched populations of study participants, and, in general, T cell-based assays, which require viably cryopreserved peripheral blood cells, are more labor and resource-intensive and can be more complex to interpret due to global HLA diversity. In order to address this gap, HIV vaccine trials would ideally measure and report the key features that define the quality of an HIV-specific T cell response (Fig. 1).

Of all these features, assessing T cell proliferative capacity and the ability to sustain killing of target cells may be the highest yield, as these qualities have been the most reliably associated with control in natural infection [21,41,42,54]. Beyond characterizing proliferation and killing capacity, key features of vaccine-elicited CD8^+^ T cells can be measured by performing deep phenotyping of vaccine-elicited HIV-specific CD8^+^ T cells by intracellular cytokine staining (ICS) and/or of MHC class I multimer staining by high-dimensional phenotyping and in-situ characterization of tissue-based vaccine-elicited CD8^+^ T cell responses. Furthermore, integrated systems immunologic assessments of cellular and plasma-based broad immune responses to different vaccine delivery systems can provide insight into the mechanisms by which each vaccine platform promotes the formation of CD8^+^ T cell responses. Capturing this comprehensive picture of vaccine-elicited CD8^+^ T cells would allow for a deeper understanding of what type of T cell response each vaccine approach can elicit, it would enable much-needed cross-platform comparisons, and it would also potentially allow for the discovery of novel correlates of protection.

## LESSONS FROM HIV CURE STUDIES

While historically most preventive vaccine approaches for HIV have focused on eliciting antibody responses, CD8^+^ T cell-based vaccines have been a more central focus of HIV cure efforts due to their potential to elicit an immune response capable of clearing established infection. Most of the qualities desired for a preventive vaccine are similar to those desired in the cure setting (e.g., high magnitude and breadth, robust proliferative and killing capacity). Although mucosal-based immune responses may be more important for prevention and lymphoid tissue-based responses are essential for cure, because HIV disseminates so rapidly across lymphoid tissues in the body after infection, preventive vaccines will also need to elicit immune responses that have the capacity to eliminate infected cells in these tissues. Similarly, therapeutic vaccines would also ideally prevent re-infection, and thus should elicit strong immunity at mucosal barriers.

Recent advances in developing CD8^+^ T cell-based vaccines for HIV cure have been extensively reviewed recently elsewhere [13,14^▪^,15^▪^,33^▪^], and vaccine designs being tested in both the prevention and cure settings are noted on Table 1. A recent study using a heterologous approach with DNA, MVA, ChAd vaccinations and a conserved mosaic insert given to people living with HIV on suppressive ART (AELIX-002) demonstrated robust T cell immunogenicity and a relationship between T cell responses and lower viral loads after ART was discontinued [129]. Data being generated from ongoing therapeutic vaccine studies with vaccines given alone or in combination with other immunotherapeutic modalities, and often with the inclusion of an ART treatment interruption, will therefore directly inform the design of studies for prevention.

## COMBINING B AND T CELL RESPONSES

As discussed at a recent NIH-sponsored meeting on ‘T and B cell synergy for HIV vaccines’, an effective vaccine strategy to prevent and/or cure HIV infection will likely require induction of both an effective antibody response (i.e., bNAbs elicited and maintained at a high titer) as well as a potent CD8^+^ T cell response. To achieve optimal B cell and CD8^+^ T cell responses, a heterologous approach may be required [7^▪▪^,^130^]. Most HIV vaccine approaches described above and listed in Table 1 do not elicit both antibodies and CD8^+^ T cell responses at a high magnitude/breadth/durability. This is in part due to the different cytokines likely required for optimal germinal center versus memory CD8^+^ T cell differentiation (i.e., IL-4 versus IL-12/IFNγ, respectively) [131]. In addition, immunogens designed to elicit Env-specific antibody responses may stimulate less effective T cell responses that target nonconserved T cell epitopes. For example, in both a prevention and therapeutic vaccine setting, inclusion of Env sequences has been shown to impair the generation of T cell responses against more conserved regions in Gag and Pol [132^▪▪^,^133^]. Going forward, it will be critical to design carefully controlled studies in humans and animal models to systematically evaluate the additive effects and trade-offs of altering vaccine platform or immunogen on the quality of both the antibody and CD8^+^ T cell response in order to understand how to elicit optimal responses in both arms.

## CONCLUSION

In recent years, newer vaccine platforms aimed at eliciting robust CD8^+^ T cell responses have been tested in the context of HIV, SARS-CoV-2, and cancer, in both preclinical and clinical settings. Going forward, we believe that addressing the following outstanding questions will be critical to move us closer to finding an optimal CD8^+^ T cell-based vaccine design for HIV prevention and/or cure:

(1) How does vaccine delivery system influence key qualities of the HIV-specific CD8^+^ T cell responses, such as magnitude (across diverse HLA types), durability, breadth of overall response and specific TCR epitope recognition, TCR avidity, polyfunctionality, proliferative and killing capacity, and homing potential?
(2) Is there a minimum breadth/number of T cell responses required to provide protection? How does immunogen design (and HLA background) affect this number?
(3) Can a single vaccine elicit mucosal-based T cell immunity and also minimize recruitment of activated CD4^+^ T cells that may be prime target cells for HIV infection?
(4) Can antibody and T cell responses synergize with one another, and are different vaccine platforms and inserts required to elicit optimal antibody versus T cell responses?

## Acknowledgements

*Figure 1* *was created with BioRender.com. Research reported in this publication was supported by the National Institute of Allergy and Infectious Diseases of the National Institutes of Health under R01AI170239, P01AI78375, and UM1AI164560 (R.L.R.), T32AI060530 (G.M.B.), and from the Bill and Melinda Gates Foundation (INV-046661, R.L.R.).*

### Financial support and sponsorship

*None.*

### Conflicts of interest

*There are no conflicts of interest.*

## REFERENCES AND RECOMMENDED READING

Papers of particular interest, published within the annual period of review, have been highlighted as:

▪ of special interest
▪▪ of outstanding interest
